# Relationship among self‐injury, experiential avoidance, cognitive fusion, anxiety, and depression in Chinese adolescent patients with nonsuicidal self‐injury

**DOI:** 10.1002/brb3.2419

**Published:** 2021-11-24

**Authors:** Zhizhong Hu, Huijuan Yu, Jingzhi Zou, Yanyan Zhang, Zihang Lu, Maorong Hu

**Affiliations:** ^1^ Department of Psychosomatic Medicine The First Affiliated Hospital of Nanchang University Nanchang Jiangxi Province China; ^2^ School of Public Administration Nanchang University Nanchang Jiangxi Province China

**Keywords:** acceptance commitment therapy, cognitive fusion, experiential avoidance, nonsuicidal self‐injury, psychological flexibility

## Abstract

**Objective:**

To explore relationship among self‐injury behavior, experiential avoidance, cognitive fusion, anxiety, and depression in Chinese adolescent patients with nonsuicidal self‐injury (NSSI).

**Methods:**

Cognitive fusion questionnaire (CFQ), Acceptance and Action Questionnaire—2nd edition (AAQ‐II), adolescent nonsuicidal self‐injury behavior questionnaire (ANSAQ), Hamilton Anxiety Scale (HAMA), and Hamilton Depression Scale (HAMD) were used as research tools to investigate 120 subjects with NSSI and 130 healthy controls.

**Results:**

The scores of CFQ and AAQ‐II in the NSSI group were significantly higher than those in the healthy control group (*p* < .001). The results of regression analysis showed that the experiential avoidance score of patients with NSSI could predict the score of self‐injury questionnaire (*β* = 0.585, *p* < .001); when predicting anxiety, only CFQ (*β* = 0.361, *p* < .001) entered the equation, with an explanatory variation of 12.3%; when predicting depression, CFQ (*β* = 0.287, *p* < .01) entered the equation, with an explanatory variation of 7.4%.

**Conclusion:**

A high level of cognitive fusion and experiential avoidance may be important factors for the maintenance of self‐injury behavior in patients with NSSI.

## Abstract 2

This study investigated the incidence of postoperative delirium in elderly patients after hip fracture surgery, and to identify risk factors for such, as part of developing a risk stratification index (RSI) system to predict a patient's risk of postoperative delirium.

## 1 INTRODUCTION

Nonsuicidal self‐injury (NSSI) is the harm done to the body when the individual has no suicidal intention. Such harm is unrecognized by social culture (Brown & Plener, 2017). Cutting wrists is the most common form of self‐injury. Conversely, tattoos, ear piercing, and smoking are not NSSI (Nock, 2009). In the fourth edition of the Diagnostic and Statistical Manual of Mental Disorders developed by the American Psychiatric Association, NSSI was once a diagnostic indicator of borderline personality disorder; however, in the newly revised fifth edition (DSM‐5), NSSI has been listed as an independent draft of disease diagnosis (Andover, 2014). Thus, NSSI has attracted increasing attention. NSSI will not only have a negative effect on individuals’ families and interpersonal relationships but even lead to death in severe cases (Mars et al., 2019).

Generally, self‐injury behavior increases rapidly in early adolescence, especially between 13 and 15 years old, peaks in middle and late adolescence, and begins to decrease in adulthood (Hawton et al., 2012). A survey showed that the lifetime prevalence rate of NSSI among teenagers in the world is 13%−17% (Swannell et al., 2014), whereas the estimated prevalence rate among Chinese adolescents (13−18 years old) is 27.4% (Han et al., 2017). China has approximately 189 million primary school students and 28 million college students in China (National Bureau of Statistics of China, 2019); hence, the number of young people with self‐injury in China may be huge. Thus, how to effectively reduce NSSI behavior is of great significance to the physical and mental health of people. At present, no unified explanation is available on the mechanism of behavioral motivation of NSSI, although it can be determined to be the result of the interaction of many factors, including biological and environmental factors (Maciejewski et al., 2014). Many studies are related to the quality–stress theory model, which holds that self‐injury behavior is caused by the interaction between adverse environment and individual susceptibility. Individuals with individual susceptibility characteristics are more likely to have self‐injury behavior. This view is supported by self‐injury theory and empirical research (Malloy‐Diniz et al., 2011). However, the meaning of the term “quality” is relatively broad, including not only physiological characteristics, such as temperament and personality, but also cognitive aspects, such as cognitive style and self‐esteem (Forrester et al., 2017). With the rise of the third generation of cognitive behavioral therapy, an increasing number of studies have focused on the cognitive level of individuals.

As one of the most representative of the third generation of cognitive behavioral therapy, acceptance commitment therapy (ACT) has received extensive attention since its establishment and has been widely used in various clinical diseases. ACT aims to help patients lead an open, flexible, and meaningful life, and this meaning is determined by the values of promoting psychological flexibility (Byrne et al., 2020). In ACT's theory, the root of all types of psychological problem is psychological inflexibility. Cognitive fusion and experience avoidance are important components of the pathological model of receiving commitment therapy (José Quintero et al., 2020), and they are important causes of psychological inflexibility. Cognitive integration indicates that the behavior of individuals is limited by their own thinking language, which makes people's behavior can only be limited by the laws of language, but not guided by the experience they have at present. Experience avoidance implies that patients will eliminate unwanted emotional experiences in various ways. Experiential avoidance and cognitive fusion belong to psychological inflexibility, which is obviously related to depression and anxiety (Hitchcock et al., 2018; Thomas & Bardeen, 2020). Depression and anxiety may also be important causes of self‐injury in patients. Studies have shown that a significant correlation exist between NSSI and depression and anxiety (Liu et al., 2021; Peters et al., 2019).

As far as NSSI is concerned, behavior is a manifestation of individual experiential avoidance and cognitive integration. Most patients with NSSI adopt self‐injury behavior to regulate negative emotions and self‐punishment (Taliaferro et al., 2019). Patients attribute some faults to themselves because of cognitive fusion and punish themselves to reduce guilt and other negative experiences through self‐injury behavior, which may be the theoretical mechanism of ACT intervention in NSSI. At present, systematic studies have investigated the intervention of ACT on NSSI. Control experiments and case studies have shown that ACT can indeed reduce individual NSSI behavior (Luoma & Villatte, 2012; Tighe et al., 2017). However, these studies are not in‐depth, and studies on the specific relationship between NSSI and experiential avoidance and cognitive fusion are limited. Therefore, the present study aims to compare the differences of experiential avoidance and cognitive fusion between patients with NSSI and healthy people in Chinese adolescents and explore the relationship between NSSI and experiential avoidance and cognitive fusion to provide more reference for ACT intervention in NSSI.

## 2 MATERIAL AND METHODS

### 2.1 Participants

The sample size needed for the study was estimated by using G‐power software, and two independent sample t‐test models were selected statistically. This study used a moderate effect dose of 0.50, a statistical test force of 1 − *β* = 0.9, and significance level of *α* = .05. On the basis of the ratio of 1:1 between the NSSI group and the control group, the results showed that at least 86 subjects were needed in each group, and 95 subjects were needed in each group according to the 10% lost follow‐up rate. A total of 120 cases in the NSSI group and 130 cases in the healthy control group were used in this study.

NSSI group: From January 2020 to December 2021 in the outpatient clinic of the first affiliated Hospital of Nanchang University, all patients with NSSI treated in the hospital were recruited and diagnosed by two senior psychiatrists according to DSM‐5 diagnostic criteria and structured interviews. The admission criteria were: (1) age 12−18 years old; (2) first visit without medication and psychotherapy; (3) self‐injury for 5 days or more in the past year; (4) self‐injury behavior in the last month; and (5) informed consent. The exclusion criteria were: (1) organic diseases, such as brain trauma; (2) intellectual disability (Wechsler intelligence test lower than 70); (3) obsessive–compulsive disorder; (4) schizophrenia; and (5) substance disorder. Finally, this study employed 120 patients with NSSI (47 were male and 73 were female; 28 were only children and 92 were non‐only children; and 76 had parents as their childhood caregivers and 44 had other people as their childhood caregivers).

Healthy control group: Subjects were mainly recruited through the Internet. The admission criteria were: (1) age 12−18 years old; (2) previous physical health; (3) no history of mental disorder; and (4) informed consent. Finally, 130 healthy subjects were enrolled in the group (48 were male and 82 were female; 43 were only children and 87 were non‐only children; 91 had parents as their childhood caregivers, and 39 had other people as their childhood caregivers).

No significant difference existed between the two groups in age, only child, and childhood caregiver variables. This study was approved by the Ethics Committee of the first affiliated Hospital of Nanchang University.

### 2.2 Measures

Research tools: (1) The adolescent nonsuicidal self‐injury behavior questionnaire (ANSAQ) (Wan et al., 2018) is divided into two parts: the behavior questionnaire and the function questionnaire. The behavior questionnaire has 12 questions, with Cronbach's *α* coefficient of .92, split‐half reliability coefficient of .85 and test–retest reliability coefficient of .84. The contribution rate of cumulative variance was 64.91%, and the correlation coefficient between functional assessment of self‐mutilation (FASM) behavior score and the behavior questionnaire was .83. The function questionnaire has 19 questions, with Cronbach's *α* coefficient of .91, split‐half reliability coefficient of .79 and test–retest reliability coefficient of .81. The contribution rate of cumulative variance was 53.9%, and The correlation coefficient between FASM behavior score and the functional questionnaire was .83. The questionnaire was scored by a five‐point Likert scale, in which 1−5 corresponded to “none, occasionally, sometimes, often, and always,” respectively. A higher score indicates a more serious self‐injury. In the current study, Cronbach's *α* coefficient of behavior questionnaire and function questionnaire were .91 and .86, respectively. The questionnaire has good reliability and validity. (2) The cognitive fusion questionnaire (CFQ) (Zhang et al., 2014) used in this study is the original questionnaire compiled by Gillanders et al. and revised in Chinese, with a total of 9 items and a score of 7. A higher the score indicates a more serious cognitive integration. The Cronbach's *α* coefficient of the questionnaire is .92, the test–retest reliability is .67, and the explanation rate of cumulative variance is 60.3%. Concurrent validity results showed that CFQ were positively correlated with total scores of Self‐rating Depression Scale (SDS) (*r* = .50, *p*< .01) and Self‐rating Anxiety Scale (SAS) (*r* = .55, *p* < .01). In the current study, the Cronbach's *α* coefficient of CFQ is .96. (3) The Acceptance and Action Questionnaire—2nd edition (AAQ‐II) (Cao et al., 2013), which comes from the Chinese scholars’ translation of the questionnaire compiled by Bond et al., has a total of 7 items with 7 grades. A higher score implies a more serious experiential avoidance. The Cronbach's *α* coefficient of the questionnaire is .88, and the test–retest reliability is .80. The explanation rate of cumulative variance is 62.5%. Concurrent validity results showed that AAQ‐II were positively correlated with total scores of SDS (*r* = .56, *p* < .01) and SAS (*r* = .55, *p* < .01). In the current study, the Cronbach's *α* coefficient of AAQ‐II is .93. (4) The Hamilton Anxiety Scale (HAMA), compiled by Hamilton, is often used to assess the anxiety severity of subjects. The scale consists of 14 items and has a 5‐grade score. A higher total score of the scale indicates a more serious anxiety of the patients. The questionnaire has good reliability and validity. (5) This study adopted a unified version of the Hamilton Depression Scale (HAMD) with 24 items and a score of 0−4. A higher score indicates a more serious depression of the patients. All the subjects were given a questionnaire by the same psychology graduate student at about 10:00 every morning, and the test was conducted in a quiet psychological evaluation room.

### 2.3 Statistical processing

The data were analyzed by SPSS 26.0 software. Multivariate analysis was used to compare the scores of social demographic characteristics, experiential avoidance, and cognitive fusion between the two groups. Independent sample *t*‐test was used to compare the scores of experiential avoidance and cognitive fusion of the subjects with different sociodemographic characteristics in the NSSI group. Correlation and regression analyses were used to explore the relationship between NSSI and experiential avoidance, cognitive fusion, anxiety, and depression. The significant level was *α* = .05.

## 3 RESULT

### 3.1 Descriptive statistics of experiential avoidance and cognitive fusion between the NSSI group and the healthy control group

The average scores of experiential avoidance and cognitive fusion in healthy control group were higher than those in NSSI group (Table 1).

**TABLE 1:** Descriptive statistics of experiential avoidance and cognitive fusion between the NSSI group and the healthy control group

Variables	Group	*N*	Mean	*s*
AAQ‐II	NSSI	120	37.9	8.4
	Control	130	21.7	8.6
CFQ	NSSI	120	49.9	10.5
	Control	130	30.6	11.1

AAQ‐II: Acceptance and Action Questionnaire—2nd edition; CFQ: Cognitive Fusion Questionnaire.

### 3.2 Multivariate analysis of AAQ‐II and CFQ on different demographic variables in two groups of subjects

The results of multivariate analysis show that only the group and childhood caregivers have main effects, while the other variables and their interactions are not significant (Table 2).

**TABLE 2:** Multivariate analysis of AAQ‐II and CFQ on different demographic variables in two groups of subjects

Source	Type III sum of squares	Df	Mean square	*F*	*p*
Dependent variables: AAQ‐II					
Corrected model	18474.8	15	1231.7	18.297	<.001
Intercept	130404.4	1	130404.4	1937.240	<.001
Group	9286.4	1	9286.4	137.956	<.001
Childhood caregiver	829.4	1	829.4	12.322	.001
Dependent variables: CFQ					
Corrected model	24992.8	15	1666.2	14.339	<.001
Intercept	231569.9	1	231569.9	1992.839	<.001
Group	12981.7	1	12981.7	111.720	<.001
Childhood caregiver	615.8	1	615.8	5.300	.022

AAQ‐II: Acceptance and Action Questionnaire—2nd edition; CFQ: Cognitive Fusion Questionnaire.

### 3.3 Comparison of experiential avoidance and cognitive fusion scores of demographic characteristics in the NSSI group

The scores of cognitive fusion and experiential avoidance of patients with NSSI were not significantly different in gender and only child variables, whereas significant differences were observed in childhood caregivers. The scores of cognitive fusion and experiential avoidance in patients with NSSI were lower than those whose caregivers were other people (Table 3).

**TABLE 3:** Comparison of experiential avoidance and cognitive fusion scores of different demographic characteristics in the NSSI group

Variables	*N*	CFQ	*t*/*F*	*p*	AAQ‐II	*t*/*F*	*p*
Gender			0.317	.752		−0.309	.758
Male	47	50.3 ± 10.9			37.6 ± 8.5		
Female	73	49.6 ± 10.3			38.1 ± 8.3		
Only child			0.440	.661		0.177	.860
Yes	28	50.6 ± 11.4			38.2 ± 8.7		
No	92	49.6 ± 10.3			37.9 ± 8.3		
Childhood caregiver			−3.386	.001		−4.840	<.001
Parents	76	47.5 ± 10.9			35.4 ± 8.0		
The others	44	54.0 ± 8.3			42.5 ± 7.0		

AAQ‐II: Acceptance and Action Questionnaire—2nd edition; CFQ: Cognitive Fusion Questionnaire.

### 3.4 Results of correlation analysis between self‐injury and anxiety, depression, cognitive fusion, and experiential avoidance

Pearson's product correlation was used to calculate the relationship among self‐injury, experiential avoidance, cognitive fusion, depression, and anxiety. The results showed that experiential avoidance was positively correlated with self‐injury (*r* = .585, *p* < .01), depression (*r* = .277, *p* < .01), and anxiety (*r* = .318, *p* < .01); and cognitive fusion was positively correlated with self‐injury (*r* = .534, *p* < .01), depression (*r* = .281, *p* < .01), and anxiety (*r* = .353, *p* < .01; Table 4).

**TABLE 4:** Correlation analysis of scores of self‐injury, anxiety, depression, cognitive fusion, and experiential avoidance in the NSSI group (*n* = 120, *r*)

Variables	ANSAQ	CFQ	AAQ‐II	HAMA	HAMD
ANSAQ	1				
CFQ	0.534**	1			
AAQ‐II	0.585**	0.790**	1		
HAMA	0.297**	0.353**	0.318**	1	
HAMD	0.246**	0.281**	0.277**	0.748**	1

ANSAQ: Adolescent Nonsuicidal Self‐Injury Behavior Questionnaire; AAQ‐II: Acceptance and Action Questionnaire—2nd edition; CFQ: Cognitive Fusion Questionnaire; HAMA: Hamilton Anxiety Scale; HAMD: Hamilton Depression Scale.

^**^ *p *< .01.

### 3.5 Regression analysis of self‐injury score, anxiety, depression, cognitive fusion, and experiential avoidance

Multiple linear regression analysis was conducted with ANSAQ total score of NSSI patients as the dependent variable, CFQ and AAQ‐II scores as predicting variables and gender, only child, and childhood caregivers as control variables. Multiple linear stepwise regression analysis was performed. AAQ‐II (*β* = 0.585) entered the equation when predicting the score of self‐injury. The level of experiential avoidance of patients with NSSI could positively predict the severity of NSSI, and the variance of explanation was 33.7%. In addition, multiple linear regression analysis was conducted with NSSI patients’ anxiety and depression score as the dependent variable, CFQ and AAQ‐II scores as predicting variables and gender, only child, and childhood caregivers as control variables. When predicting anxiety, only CFQ (*β* = 0.361) entered the equation. The level of cognitive fusion had a significant prediction and effect on anxiety in patients with NSSI, and the explanatory variance was 12.3%. When predicting depression, only CFQ (*β* = 0.287) entered the equation. The level of cognitive fusion had a significant predictive effect on depression, and its explanatory variance was 7.4%. The variance inflation factor of each regression equation was less than the index value 5, indicating that is the problem of multicollinearity did not exist among the independent variables (Table 5).

**TABLE 5:** Regression analysis of self‐injury score, anxiety and depression cognitive fusion, and experiential avoidance

Dependent variables	Predictor variables	*B*	SE	*Β*	*T*	Adjusted *R* ^2^
ANSAQ	AAQ‐II	1.094	0.140	0.585	7.834***	.337
HAMA	CFQ	0.231	0.055	0.361	4.200***	.123
HAMD	CFQ	0.331	0.102	0.287	3.250**	.074

ANSAQ: Adolescent Nonsuicidal self‐Injury Behavior Questionnaire; AAQ‐II: Acceptance and Action Questionnaire—2nd edition; CFQ: Cognitive Fusion Questionnaire; HAMA: Hamilton Anxiety Scale; HAMD: Hamilton Depression Scale.

^**^ *p* < .01.

^***^ *p* < .001.

## 4 DISCUSSION

In this study, we compared the scores of cognitive fusion and experiential avoidance between healthy subjects and NSSI patients, and studied the relationship among nonsuicidal self‐injury and cognitive fusion, experiential avoidance, anxiety, and depression. The results show that in comparison with the healthy control group, the score of each item of experiential avoidance or the total score of the NSSI group was higher than that of the healthy control group. Our results have also been confirmed in other studies on NSSI. A study of Australian undergraduate students showed that students who had a history of self‐harm scored significantly higher on experiential avoidance than those who do not have (Horgan & Martin, 2016). The experiential avoidance model suggests that NSSI has avoidance function (Chapman et al., 2006). Also, the experiential avoidance model implies that the stimuli that trigger strong negative experiences may help avoid habit changes, such as engaging in NSSI behavior. On the basis of the experiential avoidance model, NSSI is a coping strategy to regulate unwanted and/or intolerable disgust. The resulting pain relief helps maintain and strengthen the dependence on NSSI. Besides, avoidance behavior strengthened by temporarily relieving or eliminating internal imbalance, forms a self‐perpetuating cycle. Therefore, the avoidance behavior becomes the automatic escape response (Nielsen et al., 2017). In this study, some subjects said that each time when starting self‐injury, they experienced a great sense of irritability or pain, and the pleasure from cutting their wrists can help them escape those oppressive emotions.

Similarly, the results show that compared with the healthy control group, the score of each item of cognitive fusion or the total score of the NSSI group was higher than that of the healthy control group. Considerably fewer studies are available on cognitive fusion and NSSI. Some scholars believe that cognitive fusion exists in all human beings. Cognitive fusion can simplify people's cognition and connect different memories, which is beneficial to the saving of cognitive resources. However, cognitive fusion out of context is bound to cause individual behavior to be limited by thinking, and guiding behavior, and according to the experience at this moment it is impossible. Self‐injured people have a more negative coping style than ordinary people, and they have a higher degree of self‐blame and fantasy because they combine the idea of “fault” and “the reason is themselves” (Hu & Hu, 2020). And they are paranoid that nothing can be done. In this study, many subjects think that they can do nothing to change their status quo but to fight through self‐injury behavior, which also confirms the above conclusion. Patients with NSSI have higher scores of experiential avoidance and cognitive fusion, which provides a theoretical basis and possibility for ACT intervention in NSSI. NSSI can be observed not only in patients with mental and psychological problems but also in some healthy people; thus, it is not such a diagnosis as a behavioral symptom (Kiekens et al., 2019). From this perspective, as a cross‐diagnostic psychotherapy, ACT seems to have a natural fit for the intervention of NSSI. ACT also shows high integrity and acceptability in treatment, may due to ACT's different understanding of the pathology of the disease (Vakili et al., 2014). That is, the disease does not cause the pain of the patients but psychological inflexibility, including rigid cognitive fusion and the resulting avoidance strategies. Patients, through experiential avoidance, cannot effectively solve the problem because they are the root of the problem.

No significant difference was also observed in the scores of cognitive fusion and experiential avoidance between only‐child and non‐only‐child patients with NSSI. The scores of experiential avoidance of patients with NSSI were significantly different in the variables of childhood caregivers, and the scores of experiential avoidance of patients with NSSI were higher when the caregivers were other than their parents. First, when parents are busy with other matters and cannot raise their children and hand them over to others, the emotional needs of these children are often ignored. Second, children raised by others often cannot form secure attachment due to lack of parental care, and children with insecure attachment are less likely to express their emotional needs and are more inclined to adopt avoidant adjustment strategies. When in a negative emotional experience, taking self‐injurious behavior is highly likely (Kimball & Diddams, 2007). Bureau et al. (2010) reported that children who are alienated from their parents and lack parental care and protection are more likely to commit self‐injurious behavior.

The results of the correlation analysis showed a significant positive correlation between the scores of ANSAQ and the level of experiential avoidance and the level of cognitive integration, which is consistent with the existing research results. In AAQ‐II, each item reflects the avoidance and control of negative experiences by patients with NSSI, which, however, will increase the intensity and frequency of these experiences. Howe‐Martin et al. (2012) studied the relationship among alexithymia, experiential avoidance, self‐injury behavior, and suicidal ideation. The results showed that alexithymia in patients with self‐injury was significantly higher than that in normal people, and a significant correlation existed between self‐injury behavior and experiential avoidance. In CFQ, each item reflects that patients with NSSI regard the idea of self‐guilt as a fact, entangled and integrated with it, and cannot be dissociated from it; thus, a significant positive correlation exists between them. Hilt et al. (2008) found that individuals who lack cognitive resources tend to distort cognition to cope with life stress, resulting in more intense negative experience, more likely to fall into cognitive integration, form a vicious circle, and finally take self‐injuring suicide behavior. In addition, a significant positive correlation existed among NSSI, anxiety, and depression, which is consistent with previous studies. Kaur and Martin (2017) conducted a survey of 260 first‐year medical students in the University of Queensland. The results showed that the depression and anxiety of the students with self‐injury behavior increased significantly. Klimes‐Dougan et al. (2019) believed that the hypothalamic–pituitary–adrenal (HPA) axis is abnormal and may be closely related to adolescent depressive disorder and NSSI. The study suggested that the responsiveness of the HPA axis in adolescent depressive patients with NSSI is poor, such that adaptability is reduced and NSSI may occur. Bardeen studied the interaction of cognitive fusion and experiential avoidance on anxiety, depression, stress, and posttraumatic stress symptoms in a large community group and found a correlation among them (Bardeen & Fergus, 2016), which confirmed our findings.

The regression analysis results showed that only the AAQ‐II score entered the equation when predicting the total score of ANSAQ. The explanatory variation of the AAQ‐II score in ANSAQ was 29.1%, and the total score of AAQ‐II had a moderate predictive power on NSSI behavior. This finding is consistent with the research results of Nielsen et al. (2016) who found that experiential avoidance can positively predict the frequency of NSSI. According to the experiential avoidance model, the formation mechanism of NSSI is as follows. A situational event triggers an individual's aversion, and the individual implements NSSI under the interaction of many factors to escape or relieve the unpleasant emotional experience. The result of NSSI (relieving negative emotions) brings immediate satisfaction to the individual. Such negative reinforcement strengthens the connection between unpleasant emotional stimuli and NSSI behavior. Once the individual is faced with the unpleasant emotional experience again, NSSI behavior becomes an automatic escape response. Surprisingly, the total score of cognitive fusion did not enter into the equation when predicting NSSI. This result may be due to the CFQ questionnaire being based on the localization of healthy people, and its reliability and validity have not been tested in special people. Thus, the CFQ questionnaire may need to be further tested and revised. In the regression analysis of anxiety and depression of patients with NSSI, only CFQ entered the regression equation. Cognitive fusion could significantly positively predict anxiety and depression of patients with NSSI. This finding may be related to the fact that depressed people tend to identify with negative thinking and mental patterns that are extremely rigid (Mcevoy et al., 2019). Anxious patients will magnify the adverse consequences of the event, leading to cognitive fusion.

This study compares the differences in experiential avoidance and cognitive fusion between patients with NSSI and healthy people, which provides some theoretical basis for the intervention of ACT in NSSI. However, this study also has some limitations. First, this study only includes two aspects: cognitive fusion and experiential avoidance in ACT pathological model. However, the ACT pathological model includes six aspects, and the past of concept and future of fear, generalization of self, lack of value, and ineffective action are not discussed in this study. The verification of ACT pathological model in NSSI needs to be improved. Second, NSSI is not only a simple diagnostic criterion but also a symptom of many mental and psychological problems (such as depression, bipolar disorder, and borderline personality disorder). In this study, patients with NSSI are not further classified. Therefore, different mental and psychological problems with NSSI can be combined in future studies to provide a more comprehensive theoretical reference for the applicability of ACT intervention in NSSI.

## 5 CONCLUSIONS

The results of this study show that cognitive fusion and experience avoidance may be important factors in maintaining self‐injury behavior in patients with NSSI, and cognitive fusion can actively predict anxiety and depression in patients with NSSI.

## INFORMED CONSENT

All the subjects signed the informed consent form.

## FUNDING

This study was financially supported by the National Natural Science Foundation of China (81960261) and Special Funds for Postgraduates' Innovation in Jiangxi Province (YC2020‐S012).

## CONSENT FOR PUBLICATION

All authors agree to publish.

## AVAILABILITY OF DATA AND MATERIAL

All data generated or analyzed during this study are included in this published article.

### PEER REVIEW

The peer review history for this article is available at https://publons.com/publon/10.1002/brb3.2419
